# Comparing the Fagerström Test and Heaviness of Smoking Index in Predicting Smoking Abstinence in Cancer Patients

**DOI:** 10.1093/ntr/ntae120

**Published:** 2024-05-24

**Authors:** Rubén Rodríguez-Cano, George Kypriotakis, Jason D Robinson, Maher Karam-Hage, Janice A Blalock, Jennifer A Minnix, Diane Beneventi, Paul M Cinciripini

**Affiliations:** Department of Psychology, Norwegian University of Science and Technology (NTNU), Trondheim, Norway; PROMENTA Research Center, Department of Psychology, University of Oslo, Oslo, Norway; Department of Behavioral Science, University of Texas MD Anderson Cancer Center, Houston, TX, USA; Department of Behavioral Science, University of Texas MD Anderson Cancer Center, Houston, TX, USA; Department of Behavioral Science, University of Texas MD Anderson Cancer Center, Houston, TX, USA; Department of Behavioral Science, University of Texas MD Anderson Cancer Center, Houston, TX, USA; Department of Behavioral Science, University of Texas MD Anderson Cancer Center, Houston, TX, USA; Department of Behavioral Science, University of Texas MD Anderson Cancer Center, Houston, TX, USA; Department of Behavioral Science, University of Texas MD Anderson Cancer Center, Houston, TX, USA

## Abstract

**Introduction:**

People with cancer who smoke exhibit greater cigarette dependence than people without cancer who smoke, a crucial factor in smoking cessation. Research is limited on the predictive potential of the Fagerström Test for Cigarette Dependence (FTCD) and the Heaviness of Smoking Index (HSI) on smoking abstinence in cancer patients undergoing smoking cessation treatment.

**Aims and Methods:**

We analyzed data from 5934 cancer patients seeking smoking cessation treatment at The University of Texas MD Anderson Cancer Center (female 52.08%; Mean age = 55.52, SD = 11.17). We evaluated the predictive accuracy of FTCD and HSI on abstinence at 3, 6, and 9 months from the first consultation, and assessed the concordance between these tools in measuring cigarette dependence using Cohen’s kappa test and different correlation and regression models. We also analyzed variations across sex at birth and race/ethnicity.

**Results:**

Both the FTCD and the HSI demonstrated comparable predictive accuracy for smoking cessation at all follow-ups, with neither showing high accuracy (Areas Under the Curve scores around 0.6). Concordance analysis revealed substantial agreement between FTCD and HSI scores (Cohen’s kappa ~ 0.7), particularly at lower levels of dependence. However, this agreement varied by race, with reduced concordance observed in non-Hispanic Blacks.

**Conclusions:**

Our results indicate that both the FTCD and HSI are effective tools for predicting smoking cessation in cancer patients, with the HSI offering a less burdensome assessment option. Nevertheless, the findings suggest the need for tailored approaches in assessing cigarette dependence that could predict smoking cessation more accurately, considering racial differences.

**Implications:**

The burden of assessing cigarette dependence in cancer care settings can be reduced by using the HSI instead of the FTCD. In addition, both instruments could be substantially interchanged and used for meta-analytic studies examining dependence and abstinence, but race/ethnicity should be considered.

## Introduction

People with cancer who smoke are more likely to be dependent on cigarettes than people who smoke without cancer.^1^ Cigarette dependence positively correlates with increased lung cancer mortality rates,^2^ and continued smoking after a cancer diagnosis has a negative impact on the outcomes of various cancer treatments.^3^ The Fagerström Test for Cigarette Dependence (FTCD) is the “gold standard” for assessing the severity of cigarette dependence.^4,5^ The Heaviness of Smoking Index (HIS),^6^ comprised of two FTCD items (“cigarettes per day” and “time to first cigarette after waking”), is recommended for brief cigarette dependence screening in epidemiological studies.^7^ Using the HSI in cancer settings could reduce the time and burden of assessment. Yet, data are scarce on whether HSI and FTCD are equally predictive of smoking abstinence in cancer patients undergoing cessation treatment, or if they are interchangeable in this context.

In terms of predicting smoking abstinence, previous studies comparing the FTCD and HSI have shown mixed results. For example, in the general population, one study found that both the FTCD and HSI significantly predicted abstinence rates at a 6-month follow-up,^8^ but another did not at a 30-day follow-up.^9^ In clinical samples, higher dependence ranges in both FTCD and HSI showed predictive accuracy for smoking abstinence after lung cancer screening lasting up to 6 years,^2^ and in treatment-seeking smokers at 6- and 12-month follow-ups.^10^ However, a study assessing treatment-seeking smokers found that the FTCD and one item from the HSI (ie, “cigarettes per day”) did not predict abstinence at a 30-day follow-up.^11^ These inconsistencies underscore the need for further research into the predictive accuracy of these tools.

Research assessing different FTCD and HSI cutoff points to identify high cigarette dependence, have found good concordance between both instruments.^7,12^ In contrast, evaluating the concordance of the continuous measures of both instruments revealed increased discordance at higher values, indicating systematic measurement error between the FTCD and HSI.^12^ When analyzing demographic variables in concordance studies, results by sex have been mixed; one study found the same level of concordance,^12^ but another study found the FTCD to more reliably assess cigarette dependence in women than men.^13^ Racial differences in cigarette dependence are also documented,^14^ yet studies examining concordance among different races are lacking. Furthermore, no study to date has addressed the concordance of these measures among people with a cancer diagnosis who smoke.

Using a large sample of adult cancer patients seeking smoking cessation treatment (*N* = 5934), we (1) examined whether the FTCD or the HSI more accurately predicted abstinence from smoking at 3-, 6-, and 9-month post-consultation; and (2) evaluated the concordance between the FTCD and HSI in assessing cigarette dependence. We also explored how our findings might be influenced by sex at birth and racial/ethnic identification.

## Materials and Methods

### Participants

We included 5934 cancer patients seeking smoking cessation treatment at The University of Texas MD Anderson Cancer Center Tobacco Research and Treatment Program (TRTP) from September 2006 to December 2022. To be included in the analyses, patients had to attend the consultation with a TRTP clinician and complete the FTCD. This data review protocol was approved by the MD Anderson Institutional Review Board.

### Procedures

The TRTP treatment protocol started with a structured in-person consultation of 60–90 minutes, followed by 6 to 8 therapeutic sessions lasting 30–45 minutes each, over 8 to 12 weeks. Most of these sessions (95%) were conducted via telemedicine. Therefore, we relied on self-reported information to assess abstinence. However, carbon monoxide (CO) levels were obtained at all in-person visits. The congruence between 7-day point prevalence abstinence and CO levels was 93% for CO levels less than 8 ppm and 87% for CO levels less than 6 ppm. The protocol integrated behavioral counseling and pharmacotherapy, focusing on smoking cessation, and was complemented by psychological or psychiatric treatment, as needed. Details of the treatment program have been described elsewhere.^15^

### Measures

Measures, including sex, race, age, and FTCD, were administered during the initial TRTP consultation. The FTCD^4,5^ is a six-item scale with scores ranging from 0 to 10, where higher scores indicate greater dependence on cigarettes. The HSI^6^ is a subset of the FTCD, consisting of two items: “number of cigarettes per day” and “time to the first cigarette after waking,” with a scoring range from 0 to 6. We applied various cutoffs for the FTCD and HSI to categorize levels of cigarette dependence, in accordance with classifications commonly documented in the literature^7,12^ (Table S1).

### Statistical Analyses

To assess the predictive accuracy of the baseline FTCD and HSI on smoking abstinence at 3, 6, and 9 months, we used logistic regression with abstinence as the dependent variable. Abstinence was defined as complete cessation, without a single puff, in the 7 days preceding each follow-up. We used intention-to-treat abstinence, coding missing data as smoking. Sensitivity analyses including responders only suggested no differences in baseline measures nor in the prediction of abstinence by the two dependence instruments (Tables S2 and S3). We provided odds ratios (ORs) and adjusted ORs (aORs) for continuous and cutoff measures, with adjustments made for sex, race, and age. For assessing the models’ predictive accuracy and goodness-of-fit, we applied the Akaike Information Criterion, the Bayesian Information Criterion (BIC)—where lower values indicate a better model fit—and the Brier Score for the accuracy of probabilistic predictions. Furthermore, we calculated the Area Under the Receiver Operating Characteristic Curve (AUC-ROC) to evaluate the model’s discriminative ability between individuals who maintained abstinence and those who did not. Previous studies indicated that the time to first cigarette item (TTFC) is an indicator of cigarette dependence and predicts cessation outcomes.^10,16^ Thus, we also analyze the predictive value of TTFC on smoking abstinence at 3-, 6-, and 9-month follow-ups (Tables S3 and S4).

To evaluate the concordance between the FTCD and HSI, we calculated Cohen’s kappa coefficient, agreement percentage, and expected agreement percentage. Following Landis and Koch’s benchmarks,^17^ a kappa coefficient above 0.80 signifies excellent agreement, 0.61–0.80 suggests substantial to moderate agreement, and values below 0.40 indicate poor to fair agreement. We employed bootstrap methods to determine confidence intervals for kappa and computed Pearson and Spearman correlation coefficients to examine linear relationships, respectively. We also applied linear regression analysis to model the relationship between FTCD and HSI scores. In addition, we used the correlation-concordance coefficient of Lin^18^ and used Bland-Altman plots to detect any systematic bias and to visually assess agreement. To adjust for errors-in-variable bias, we performed Deming regression.^19^ We conducted analyses with the global sample, and by sex at birth and racial groups. Analyses were conducted using STATA version 18.^20^

## Results

Demographics, cigarette dependence levels, and abstinence rates are displayed in Table S5.

### Abstinence Predictive Accuracy

Continuous FTCD and HSI scores consistently outperformed categorical scores in predictive accuracy, with lower Akaike Information Criterion/BIC values and slightly higher ORs and AUROC scores at 9-month follow-up (AUROC 0.56 and 0.57 for FTCD and HSI, respectively; Table 1).

**Table 1.** Predictive Accuracy of the FTCD and HSI on Smoking Abstinence at 9-Months Follow-up (*N* = 5934)

	*N*	OR	OR 95%CI	AOR	AOR 95%CI	AIC	BIC	Brier score	ROC area
*Continue measures*									
FTCD	5934	0.90	0.89 to 0.92	0.90	0.87 to 0.91	7374.07	7387.44	0.22	0.56
HSI	5934	0.85	0.82 to 0.88	0.84	0.81 to 0.87	7366.81	7380.19	0.21	0.57
Categorical measures									
3 groups									
*FTCD*									
FTCD low	2106	1.45	1.29 to 1.62	1.48	1.32 to 1.66	7400.08	7413.46	0.22	0.54
FTCD moderate	3368	0.77	0.69 to 0.86	0.76	0.68 to 0.84	7420.02	7433.40	0.22	0.53
FTCD high	460	0.72	0.58 to 0.90	0.72	0.58 to 0.89	7433.48	7446.86	0.22	0.51
*HSI*									
HSI low	1265	1.64	1.44 to 1.86	1.67	1.46 to 1.90	7386.70	7400.08	0.22	0.54
HSI moderate	4024	0.77	0.69 to 0.87	0.77	0.69 to 0.87	7423.59	7436.97	0.22	0.53
HSI high	645	0.72	0.60 to 0.87	0.71	0.59 to 0.85	7430.24	7443.62	0.22	0.51
** **2 groups									
*FTCD*									
FTCD ≥ 6 high	1803	0.69	0.61 to 0.78	0.68	0.60 to 0.77	7406.72	7420.09	0.22	0.54
FTCD ≥ 8 high	460	0.72	0.58 to 0.90	0.72	0.58 to 0.89	7433.48	7446.86	0.22	0.51
*HSI*									
HSI ≥ 4 High	1783	0.67	0.60 to 0.76	0.66	0.58 to 0.75	7402.19	7415.56	0.22	0.54
HSI ≥ 5 high	645	0.72	0.60 to 0.87	0.71	0.59 to 0.86	7430.24	7443.62	0.22	0.51

OR = Odd Ration; CI = Confidence Intervals; AOR = adjusted OR by age, sex, and race; AIC = Akaike Information Criterion; BIC = Bayesian Information Criterion; ROC = receiver operating characteristic curve.

FTCD = Fagerström Test for Cigarette Dependence (low ≤ 3, moderate 4–7, high ≥ 8); HSI = heaviness of smoking index (low 0–1, moderate 2–4, high 5–6).

When comparing the categorical FTCD and HSI, both measures exhibited comparable predictive power for smoking abstinence at the 9-month follow-up (Table 1). This is reflected in the overlapping ranges of AUROC (0.51 to 0.54) and Brier scores = 0.22, suggesting that either measure can be used interchangeably for prediction purposes with no significant loss of predictive accuracy.

The 3- and 6-month follow-up results are similar to those of the 9-month follow-up (Tables S6 and S7).

Results show that TTFC has a lower predictive value at 3- and 9-month follow-ups compared to FTCD (Tables 1, Tables S4, S6, And S7). At 6 months, despite FTCD having higher ORs (OR = 0.89, 95% CI = 0.86 to 0.91), the confidence interval for TTFC’s adjusted ORs overlaps with that of FTCD (OR = 0.82, 95% CI = 0.78 to 0.87). This overlap indicates no significant difference between the continuous measures of both instruments when adjusting for all covariates at the 6-month follow-up. Compared to HSI, TTFC shows no significant difference in predictive values for abstinence across all follow-ups.

### Concordance Analyses

#### Categorical (Cutoffs)

The concordance varied between the FTCD and the HSI across different combinations and demographic groups, as detailed in Table S8. The highest kappa coefficient of 0.71 (Bootstrap CI: 0.70 to 0.73), which indicates a substantial agreement between the FTCD and HSI, was observed in combination 4, where the FTCD score was six or more and the HSI score was four or more in the overall sample. The analysis by sex at birth revealed a slightly higher kappa value for females in Combination 4, registering at 0.72 (Bootstrap CI: 0.70 to 0.75), compared to males, who had a kappa of 0.70 (Bootstrap CI: 0.67 to 0.73). However, this difference is not meaningful since CI overlaps among both sex groups.

In terms of race/ethnicity, the concordance between FTCD and the HSI showed notable variations (Table S9). Combination 4 also yielded the highest kappa values for all racial groups except non-Hispanic Blacks. For example, non-Hispanic Whites had a kappa of 0.72 (Bootstrap CI: 0.71 to 0.75), and Hispanics (of any race) exhibited a kappa of 0.72 (Bootstrap CI: 0.62 to 0.82). Blacks, however, showed a peak kappa value of 0.65 in Combination 3 but similar values to those in Combination 4 of 0.56. Asian- and Native Americans had limited data in some combinations, which led to kappa values not being computed or displaying high variability.

#### Continuous Total Scores

The analyses of the continuous total score are displayed in Tables S10 and S11. We found a strong Pearson correlation (*r* = 0.89) between FTCD and HSI, consistent across sex and varying slightly by ethnicity. Non-Hispanic Blacks had a slightly lower correlation (*r* = 0.87), while Asian-Americans and Native Americans showed more variability (*r* = 0.85 and 0.91, respectively). Similar trends were observed in Spearman’s rank correlation, with r values ranging from 0.86 to 0.89 across ethnic groups. Lin’s concordance coefficient indicated moderate global concordance (ρ_c_ = 0.61), with the lowest concordance in Asian-Americans (ρ_c_ = 0.51) and the highest in Hispanics of any race (ρ_c_ = 0.64). Regression analysis revealed similar coefficients across sex and ethnicity (b = 0.89–0.91), with the lowest value for Asian-Americans (b = 0.85).

**Figure 1.**
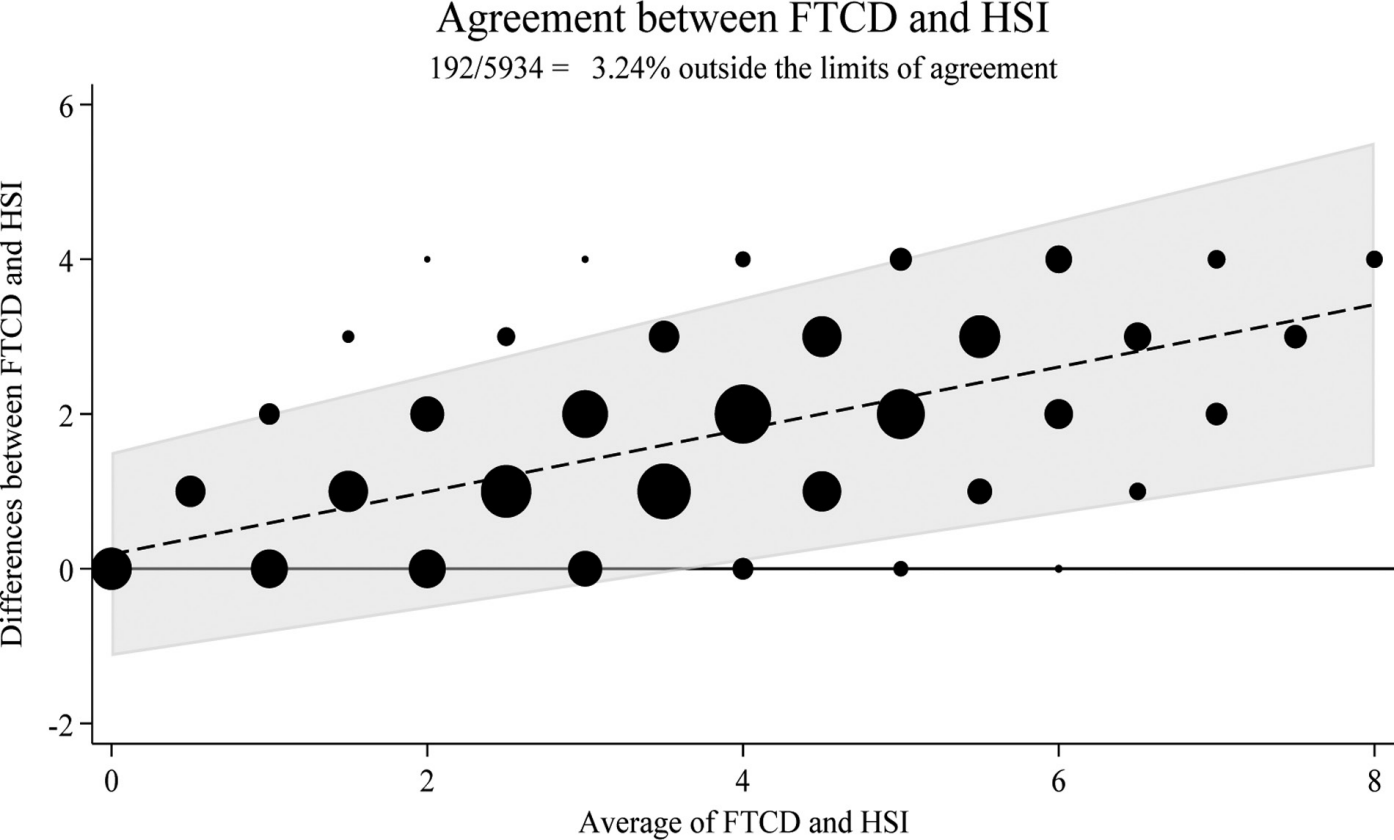
Bland-Altman plot assessing agreement between Fagerström Test for Cigarette Dependence (FTCD) and Heaviness of Smoking Index (HSI) scores. The dashed line represents the mean difference between FTCD and HSI. The shaded area depicts the 95% limits of agreement. Data point size correlates with the number of cases, indicating variability in the differences between the two measures. A solid line at axis-y = 0 indicates no difference between both measures.

Bland-Altman plot analyses showed marginal sex differences in agreement (percentage outside the limits 4.85% for males, 3.72% for females) and greater variation by race, with non-Hispanic Blacks showing the least agreement (Figures 1, Figures S1, and S2). Largely, both overall and within individual groups, the level of disagreement between the two measures tends to escalate as the average scores for FTCD and HSI increased. Finally, Deming regression analyses indicated variations in intercepts and FTCD coefficients across demographics, indicating that the HSI mean values differed among groups when the FTCD was 0. Deming regression slopes suggested stable relationships between the FTCD and HSI across all groups.

## Discussion

The current study evaluated the predictive accuracy of abstinence and concordance between the FTCD and the HSI among treatment-seeking smokers with a cancer diagnosis. Similar to previous research,^8,10^ our results show that both the FTCD and HSI measured at baseline have comparable predictive accuracy for smoking cessation at 3-, 6-, and 9-month post-consultation. In line with prior findings,^10^ neither instrument demonstrated high accuracy in predicting smoking abstinence, with AUROC scores around 60. Considering their similar predictive accuracy, the HSI’s shorter format may be preferable in clinical studies of smoking cessation among cancer patients when a cut-off for cigarette dependence is needed. If there is interest in using only a continuous measure for cigarette dependence, the one-item TTFC may be preferable to HSI, as it shows similar predictive accuracy across all follow-ups.

Consistent with other research,^7,12^ our study found substantial concordance between the FTCD and HSI in assessing cigarette dependence, particularly when HSI scores were 4 or more and FTCD scores were 6 or more (Cohen’s kappa~0.70). However, a continuous measures analysis, similar to what was found in treatment-seeking smokers without cancer,^12^ indicated decreased agreement at higher mean levels of the FTCD and the HSI, suggesting optimal agreement at lower dependence levels. Our study observed similar concordance across sexes, in contrast with some studies,^13^ but consistent with others.^12^ A novel aspect of our study was the analysis of concordance across different racial groups in people who smoke and have cancer. All racial groups exhibited substantial agreement; however, concordance was notably lower among Non-Hispanic Blacks. This reduction may be attributed to variations in how nicotine metabolic rates correlate with different measures of cigarette dependence. Specifically, in a previous study in Non-Hispanic Blacks nicotine metabolic rates were primarily linked to physiological measures of cigarette use, as evidenced by HSI items, rather than the behavioral components assessed by the FTCD.^14^ This suggests that the interchangeability of the FTCD and HSI in assessing cigarette dependence may be limited in this group.

Our study has some limitations. Firstly, while our findings align with those in the general population and among treatment-seeking smokers, they are specific to cancer patients who are treatment-seeking smokers and do not include those who smoke but are not motivated to quit. Secondly, we focused only on medium-term abstinence (up to 9 months); hence, future research should explore the predictive accuracy of both instruments over longer-term follow-ups. Secondly, our reliance on self-reported abstinence, due to phone-based treatment and follow-ups, might reduce the accuracy of our findings. However, around 90% congruence between self-reported abstinence and carbon monoxide levels has been reported among cancer patients.^15^ Lastly, we used point prevalence abstinence rather than continuous abstinence. Future studies should also include continuous abstinence since it is a better predictor for long-term abstinence.

In conclusion, our study demonstrates the comparable predictive accuracy of the FTCD and the HSI for smoking cessation among cancer patients. Specifically, the two-item HSI showed accuracy similar to that of the FTCD in predicting abstinence, offering a less burdensome option for cigarette dependence assessment among smoking cessation treatment-seeking smokers with cancer, especially when a cutoff for cigarette dependence is used. Comparisons between HSI and the one-item TTFC suggest similar predictive accuracy for abstinence; thus, TTFC presents an even less burdensome option for assessing cigarette dependence than HSI. However, our findings are limited to treatment-seeking smokers with cancer, indicating the need for broader research, especially in epidemiological studies involving cancer patients who smoke and are not motivated to quit. Moreover, the variability in predictive accuracy across different races underscores the need for personalized smoking dependence assessment by racial/ethnicity group. This study contributes to the field by highlighting the utility of a shorter assessment tool that can be used to assess cigarette dependence within the context of smoking cessation treatment in cancer care.

## Supplementary Material

Supplementary material is available at *Nicotine and Tobacco Research* online.

ntae120_suppl_Supplementary_Material

## Data Availability

Due to the protection of patient health information (PHI), data sharing is not possible for this study.
