# Supplementary material for: Comparing the Fagerström Test and Heaviness of Smoking Index in Predicting Smoking Abstinence in Cancer Patients
Source: Nicotine Tob Res. 2024 May 24;26(11):1576–81. doi: 10.1093/ntr/ntae120 (PMC11494472; doi:10.1093/ntr/ntae120)
Supplement: ntae120_suppl_Supplementary_Material [file ntae120_suppl_supplementary_material.docx]

**Supplementary materials:**

**Comparing the Fagerström Test and Heaviness of Smoking Index in Predicting Smoking Abstinence in Cancer Patients**

Running title: Fagerström Test for Dependence and the Heaviness of Smoking Index

Rubén Rodríguez-Cano, PhD^1,2*^, George Kypriotakis, PhD ^3^, Jason D. Robinson, PhD ^3^, Maher Karam‐Hage, MD^3^, Janice A. Blalock PhD ^3^, Jennifer A. Minnix, PhD ^3^, Diane Beneventi, PhD ^3^ and Paul M. Cinciripini PhD ^3^.

^1^Department of Psychology, Norwegian University of Science and Technology (NTNU), Trondheim, Norway.

^2^PROMENTA Research Center, Department of Psychology, University of Oslo, Oslo, Norway.

^3^Department of Behavioral Science, The University of Texas MD Anderson Cancer Center, Houston, TX, USA.

*Corresponding author

Department of Psychology, Norwegian University of Science and Technology (NTNU),

NO-7491, Trondheim, Norway.

E-Mail: ruben.cano@ntnu.no

Table s1. Cigarette dependence level by Fagerström Test for Cigarette Dependence (FTCD) and Heaviness of Smoking Index (HSI).

| **Instrument and cut-off categories** | **Scores** |
| --- | --- |
| Three-categories cut-off |  |
| *FTCD* |  |
| FTCD low | 0-3 |
| FTCD moderate | 4-7 |
| FTCD high | 8-10 |
| *HSI* |  |
| HSI low | 0-1 |
| HSI moderate | 2-4 |
| HSI high | 5-6 |
| Two-categories cut-off |  |
| *FTCD* |  |
| FTCD low-moderate | 0-5 |
| FTCD high | 6-10 |
| *FTCD* |  |
| FTCD low-moderate | 0-7 |
| FTCD high | 8-10 |
| *HSI* |  |
| HSI low-moderate | 0-3 |
| HSI high | 4-6 |
| *HSI* |  |
| HSI low-moderate | 0-4 |
| HSI high | 5-6 |

Table s2. Sample characteristics at baseline by attendance to follow-up (N = 5,934).

|  | ***3-months follow-up*** | | | | | ***6-months follow-up*** | | | | | ***9-months follow-up*** | | | | |
| --- | --- | --- | --- | --- | --- | --- | --- | --- | --- | --- | --- | --- | --- | --- | --- |
|  | **Attend**  **(n = 4820)** | | **Missing**  **(n = 1114)** | |  | **Attend**  **(n = 4462)** | | **Missing**  **(n = 1472)** | |  | **Attend**  **(n = 4007)** | | **Missing**  **(n = 1927)** | |  |
| **Variable** | N/Mean | %/SD | N/Mean | %/SD | X2/t(df) | N/Mean | %/SD | N/Mean | %/SD | X2/F(df) | N/Mean | %/SD | N/Mean | %/SD | X2/F(df) |
| Age | 55.59 | 11.16 | 55.21 | 11.22 | 1.04 (5932) | 55.63 | 11.05 | 55.18 | 11.51 | 1.33 (5932) | 55.74 | 11.16 | 55.04 | 11.22 | 2.26 (5932)* |
| Female | 2531 | 52.51 | 560 | 50.27 | 1.82 (1) | 2334 | 52.31 | 757 | 51.43 | 0.34 (1) | 2078 | 51.86 | 1013 | 55.57 | 0.26 (1) |
| Race |  |  |  |  | 9.18(5) |  |  |  |  | 10.70(5) |  |  |  |  | 18.50(5)** |
| White | 3771 | 78.24 | 864 | 77.56 |  | 3490 | 78.22 | 1145 | 77.79 |  | 3156 | 78.76 | 1479 | 76.75 |  |
| Black | 586 | 12.16 | 123 | 11.04 |  | 548 | 12.28 | 161 | 10.94 |  | 490 | 12.23 | 219 | 11.36 |  |
| Hispanic | 299 | 6.20 | 73 | 6.55 |  | 274 | 6.14 | 98 | 6.66 |  | 240 | 5.99 | 132 | 6.85 |  |
| Asian | 46 | 0.95 | 10 | 0.90 |  | 43 | 0.96 | 13 | 0.88 |  | 32 | 0.80 | 24 | 1.25 |  |
| Native American | 12 | 0.25 | 6 | 0.54 |  | 10 | 0.22 | 8 | 0.54 |  | 8 | 0.20 | 10 | 0.52 |  |
| Other | 106 | 2.20 | 38 | 3.41 |  | 97 | 2.17 | 47 | 3.19 |  | 81 | 2.02 | 63 | 3.27 |  |
| FTCD | 4.33 | 2.21 | 4.33 | 2.20 | 0.13(5932) | 4.30 | 2.20 | 4.42 | 2.22 | 0.13(5932) | 4.29 | 2.22 | 4.41 | 2.17 | -1.78(5932) |
| HSI | 2.73 | 1.50 | 2.68 | 1.50 | 1.12(5932) | 2.72 | 1.49 | 2.72 | 1.50 | 0.09(5932) | 2.71 | 1.50 | 2.72 | 1.50 | -0.43(5932) |
| TTFC | 1.88 | 0.96 | 1.86 | 0.86 | 0.45(5932) | 1.87 | 0.96 | 1.88 | 0.97 | -0.28(5932) | 1.86 | 0.96 | 1.89 | 0.95 | -1.12(5932) |

*Note.* FTCD = Fagerström Test for Cigarette Dependence; HSI = Heaviness of Smoking Index; TTFC = Time to first cigarette.

* p < 0.05 ** p< 0.01

Table s3. Predictive accuracy of the Fagerström Test for Cigarette Dependence (FTCD), Heaviness of Smoking Index (HSI) and time to first cigarette (TTFC) on smoking abstinence among those responders only.

|  | N | OR | OR  95%CI | AOR | AOR  95%CI | AIC | BIC | Brier  score | ROC area |
| --- | --- | --- | --- | --- | --- | --- | --- | --- | --- |
| *3-months follow-up* |  |  |  |  |  |  |  |  |  |
| FTCD | 4820 | 0.85 | 0.83-0.87 | 0.84 | 0.82-0.86 | 6527.02 | 6539.98 | 0.24 | 0.60 |
| HSI | 4820 | 0.79 | 0.76-0.82 | 0.77 | 0.74-0.80 | 6528.67 | 6541.63 | 0.24 | 0.60 |
| TTFC | 4820 | 0.74 | 0.70-0.79 | 0.74 | 0.70-0.79 | 6579.54 | 6592.50 | 0.24 | 0.58 |
| *6-months follow-up* |  |  |  |  |  |  |  |  |  |
| FTCD | 4462 | 0.87 | 0.85-0.90 | 0.87 | 0.84-0.89 | 6095.42 | 6108.23 | 0.24 | 0.58 |
| HSI | 4462 | 0.81 | 0.78-0.84 | 0.80 | 0.77-0.83 | 6084.08 | 6096.88 | 0.24 | 0.59 |
| TTFC | 4462 | 0.79 | 0.74-0.84 | 0.78 | 0.74-0.84 | 6131.64 | 6144.45 | 0.25 | 0.56 |
| *9-months follow-up* |  |  |  |  |  |  |  |  |  |
| FTCD | 4007 | 0.86 | 0.86-0.91 | 0.88 | 0.86-0.91 | 5477.48 | 5490.07 | 0.25 | 0.57 |
| HSI | 4007 | 0.82 | 0.78-0.85 | 0.80 | 0.77-0.84 | 5455.91 | 5468.51 | 0.24 | 0.58 |
| TTFC | 4007 | 0.79 | 0.74-0.85 | 0.79 | 0.74-0.84 | 5498.85 | 5511.44 | 0.25 | 0.56 |

*Note.* OR = Odd Ration; CI= Confidence Intervals; AOR = adjusted OR by age, sex, and race; AIC = Akaike Information Criterion; BIC = Bayesian Information Criterion; ROC= receiver operating characteristic curve.

Table s4. Predictive accuracy of the time to first cigarette (TTFC) on smoking abstinence (N = 5,934).

|  | N | OR | OR  95%CI | AOR | AOR  95%CI | AIC | BIC | Brier  score | ROC area |
| --- | --- | --- | --- | --- | --- | --- | --- | --- | --- |
| *3-months follow-up* |  |  |  |  |  |  |  |  |  |
| TTFC | 5934 | 0.78 | 0.74-0.82 | 0.78 | 0.74-0.82 | 7855.63 | 7869.01 | 0.23 | 0.56 |
| *6-months follow-up* |  |  |  |  |  |  |  |  |  |
| TTFC | 5934 | 0.83 | 0.78-0.87 | 0.82 | 0.78-0.87 | 7819.03 | 7832.41 | 0.23 | 0.55 |
| *9-months follow-up* |  |  |  |  |  |  |  |  |  |
| TTFC | 5934 | 0.82 | 0.80-0.87 | 0.82 | 0.78-0.87 | 7397.83 | 7411.21 | 0.22 | 0.55 |

*Note.* OR = Odds Ratio; CI= Confidence Intervals; AOR = adjusted OR by age, sex, and race; AIC = Akaike Information Criterion; BIC = Bayesian Information Criterion; ROC= receiver operating characteristic curve.

Table s5. Total sample characteristics by sex and race (N = 5,934).

| **Variable** | ***N/Mean*** | ***%/SD*** |
| --- | --- | --- |
| Age | 55.52 | 11.17 |
| Female | 3091 | 52.08 |
| Race |  |  |
| White, Non-Hispanic | 4635 | 78.11 |
| Black, Non-Hispanic | 709 | 11.95 |
| Hispanic, Any Race | 372 | 6.27 |
| Native American, Non-Hispanic | 56 | 0.94 |
| Asian, Non-Hispanic | 18 | 0.31 |
| N/A or Other | 144 | 2.42 |
| FTCD total | 4.33 | 2.20 |
| HSI total | 2.72 | 1.50 |
| Abstinence^a^ |  |  |
| 3-month follow-up | 2308 | 38.89 |
| 6-month follow-up | 2236 | 37.68 |
| 9-month follow-up | 1898 | 31.98 |

*Note.* FTCD = Fagerström Test for Cigarette Dependence; HSI = Heaviness of Smoking Index;

^a^The original publication reported Intention-to-Treat abstinence rates of 41.4%, 39.9%, and 36.5% at 3-, 6-, and 9-month follow-ups, respectively^[[1]](#footnote-2)^. In our study, the abstinence rates may differ because our analysis accounts for patients' first participation in the smoking cessation program, whereas the original study assessed the patients' most recent enrollment in the program.

Table s6. Predictive accuracy of the Fagerström Test for Cigarette Dependence (FTCD) and Heaviness of Smoking Index (HSI) on smoking abstinence at 3-months follow-up (N = 5,934).

|  | N | OR | OR  95%CI | AOR | AOR  95%CI | AIC | BIC | Brier  score | ROC area |
| --- | --- | --- | --- | --- | --- | --- | --- | --- | --- |
| *3-months follow-up* |  |  |  |  |  |  |  |  |  |
| **Continue measures** |  |  |  |  |  |  |  |  |  |
| FTCD | 5934 | 0.87 | 0.85-0.89 | 0.86 | 0.84-0.88 | 7808.30 | 7821.68 | 0.23 | 0.59 |
| HSI | 5934 | 0.82 | 0.79-0.85 | 0.81 | 0.78-0.84 | 7818.75 | 7832.13 | 0.23 | 0.58 |
| **Categorical measures** |  |  |  |  |  |  |  |  |  |
| **3 groups** |  |  |  |  |  |  |  |  |  |
| *FTCD* |  |  |  |  |  |  |  |  |  |
| FTCD low | 2106 | 1.87 | 1.68-2.07 | 1.78 | 1.59-2.00 | 7835.62 | 7849.00 | 0.23 | 0.57 |
| FTCD moderate | 3368 | 0.55 | 0.50-0.61 | 0.66 | 0.59-0.74 | 7880.19 | 7893.57 | 0.24 | 0.55 |
| FTCD high | 460 | 0.65 | 0.52-0.69 | 0.64 | 0.52-0.79 | 7917.08 | 7930.45 | 0.24 | 0.51 |
| *HSI* |  |  |  |  |  |  |  |  |  |
| HSI low | 1265 | 1.77 | 1.56-2.00 | 1.81 | 1.59-2.05 | 7855.99 | 7869.36 | 0.23 | 0.55 |
| HSI moderate | 4024 | 0.76 | 0.69-0.86 | 0.77 | 0.69-0.86 | 7913.30 | 7926.67 | 0.24 | 0.53 |
| HSI high | 645 | 0.65 | 0.62-0.70 | 0.63 | 0.52-0.75 | 7911.44 | 7924.92 | 0.24 | 0.52 |
| **2 groups** |  |  |  |  |  |  |  |  |  |
| *FTCD* |  |  |  |  |  |  |  |  |  |
| FTCD>=6 High | 1803 | 0.63 | 0.56-0.70 | 0.62 | 0.55-0.69 | 7873.62 | 7887.00 | 0.24 | 0.55 |
| FTCD>=8 High | 460 | 0.64 | 0.50-0.79 | 0.64 | 0.52-0.79 | 7917.08 | 7930.45 | 0.24 | 0.51 |
| *HSI* |  |  |  |  |  |  |  |  |  |
| HSI>=4 High | 1783 | 0.62 | 0.55-0.70 | 0.60 | 0.53-0.67 | 7869.21 | 7882.59 | 0.23 | 0.55 |
| HSI>=5 High | 645 | 0.65 | 0.63-0.70 | 0.62 | 0.53-0.75 | 7911.54 | 7924.92 | 0.24 | 0.52 |

*Note.* OR = Odd Ration; CI= Confidence Intervals; AOR = adjusted OR by age, sex, and race; AIC = Akaike Information Criterion; BIC = Bayesian Information Criterion; ROC= receiver operating characteristic curve.

FTCD = Fagerström Test for Cigarette Dependence (Low <= 3, Moderate 4–7, High>=8); HSI = Heaviness of Smoking Index (Low 0–1, Moderate 2–4, High 5–6).

Table s7. Predictive accuracy of the Fagerström Test for Cigarette Dependence (FTCD) and Heaviness of Smoking Index (HSI) on smoking abstinence at 6-months follow-up (N = 5,934).

|  | N | OR | OR  95%CI | AOR | AOR  95%CI | AIC | BIC | Brier  score | ROC area |
| --- | --- | --- | --- | --- | --- | --- | --- | --- | --- |
| *6-months follow-up* |  |  |  |  |  |  |  |  |  |
| **Continue measures** |  |  |  |  |  |  |  |  |  |
| FTCD | 5934 | 0.89 | 0.87-0.81 | 0.89 | 0.86-0.91 | 7776.89 | 7790.27 | 0.23 | 0.57 |
| HSI | 5934 | 0.85 | 0.82-0.88 | 0.84 | 0.81-0.87 | 7783.31 | 7796.69 | 0.23 | 0.57 |
| **Categorical measures** |  |  |  |  |  |  |  |  |  |
| **3 groups** |  |  |  |  |  |  |  |  |  |
| *FTCD* |  |  |  |  |  |  |  |  |  |
| FTCD low | 2106 | 1.50 | 1.34-1.67 | 1.52 | 1.36-1.70 | 7813.39 | 7826.76 | 0.23 | 0.55 |
| FTCD moderate | 3368 | 0.76 | 0.69-0.85 | 0.76 | 0.68-0.84 | 7841.58 | 7854.95 | 0.23 | 0.53 |
| FTCD high | 460 | 0.67 | 0.54-0.82 | 0.64 | 0.52-0.79 | 7851.10 | 7864.48 | 0.23 | 0.51 |
| *HSI* |  |  |  |  |  |  |  |  |  |
| HSI low | 1265 | 1.57 | 1.38-1.78 | 1.58 | 1.39-1.80 | 7817.87 | 7831.25 | 0.23 | 0.54 |
| HSI moderate | 4024 | 0.83 | 0.74-0.92 | 0.83 | 0.74-0.93 | 7855.22 | 7868.59 | 0.23 | 0.52 |
| HSI high | 645 | 0.68 | 0.57-0.82 | 0.67 | 0.56-0.80 | 7848.01 | 7861.38 | 0.23 | 0.52 |
| **2 groups** |  |  |  |  |  |  |  |  |  |
| *FTCD* |  |  |  |  |  |  |  |  |  |
| FTCD>=6 High | 1803 | 0.65 | 0.56-0.73 | 0.64 | 0.57-0.72 | 7812.94 | 7826.32 | 0.23 | 0.54 |
| FTCD>=8 High | 460 | 0.67 | 0.54-0.82 | 0.67 | 0.54-0.82 | 7851.10 | 7864.48 | 0.23 | 0.51 |
| *HSI* |  |  |  |  |  |  |  |  |  |
| HSI>=4 High | 1783 | 0.63 | 0.56-0.71 | 0.61 | 0.55-0.69 | 7806.78 | 7820.16 | 0.23 | 0.55 |
| HSI>=5 High | 645 | 0.69 | 0.58-0.82 | 0.67 | 0.56-0.80 | 7890.01 | 7861.37 | 0.23 | 0.52 |

*Note.* OR = Odd Ration; CI= Confidence Intervals; AOR = adjusted OR by age, sex, and race; AIC = Akaike Information Criterion; BIC = Bayesian Information Criterion; ROC= receiver operating characteristic curve.

FTCD = Fagerström Test for Cigarette Dependence (Low <= 3, Moderate 4–7, High>=8); HSI = Heaviness of Smoking Index (Low 0–1, Moderate 2–4, High 5–6).

Table s8. Concordance values using Cohen’s kappa index and agreement percentage for different combinations of the Fagerström Test for Cigarette Dependence (FTCD) and Heaviness of Smoking Index (HSI) by sex.

| **Combination** | **Group** | **n** | **Agreement (%)** | **Expected agreement (%)** | **Kappa (-1,1)** | **SE** | **Bootstrap CI^a^** |
| --- | --- | --- | --- | --- | --- | --- | --- |
| 1 | Total | 5934 | 88.64 | 70.51 | 0.57 | 0.014 | 0.55-0.60 |
|  | Male | 2843 | 88.64 | 70.56 | 0.57 | 0.014 | 0.55-0.60 |
|  | Female | 3091 | 88.58 | 70.84 | 0.57 | 0.013 | 0.54-0.60 |
| 2 | Total | 5934 | 77.70 | 66.86 | 0.33 | 0.010 | 0.31-0.35 |
|  | Male | 2843 | 75,84 | 65,17 | 0.31 | 0.014 | 0.27-0.34 |
|  | Female | 3091 | 79.42 | 68.42 | 0.35 | 0.014 | 0.32-0.39 |
| 3 | Total | 5934 | 93.07 | 83.06 | 0.59 | 0.013 | 0.55-0.63 |
|  | Male | 2843 | 92.16 | 81.54 | 0.58 | 0.018 | 0.52-0.63 |
|  | Female | 3091 | 93.92 | 84.47 | 0.61 | 0.018 | 0.56-0.66 |
| 4 | Total | 5934 | 87.87 | 57.83 | 0.71 | 0.013 | 0.70-0.73 |
|  | Male | 2843 | 86.92 | 56.83 | 0.70 | 0.019 | 0.67-0.73 |
|  | Female | 3091 | 88.74 | 58.79 | 0.73 | 0.018 | 0.70-0.75 |
| 5 | Total | 5934 | 79.54 | 65.35 | 0.41 | 0.012 | 0.39-0.44 |
|  | Male | 2843 | 80.16 | 64.24 | 0.45 | 0.016 | 0.41-0.48 |
|  | Female | 3091 | 78.79 | 66.45 | 0.37 | 0.014 | 0.34-0.41 |

*Note.* CI= Confidence Intervals; ^a^Bias corrected with 1000 samples.

1. HSI Low 0–1, Moderate 2–4, High 5–6 vs. FTCD Low <= 3, Moderate 4–7, High>=8.

2. HSI Low–Moderate <4, High>=4 versus FTCD Low–Moderate<8, High>=8.

3. HSI Low–Moderate<=4, High>4 versus FTCD Low–Moderate<8, High>=8.

4. HSI Low–Moderate<4, High>=4 versus FTCD Low–Moderate<6, High>=6.

5. HSI Low–Moderate<=4, High>4 versus FTCD Low–Moderate<6, High>=6.

Table s9. Concordance values using Cohen’s kappa index and agreement percentage for different combinations of the Fagerström Test for Cigarette Dependence (FTCD) and Heaviness of Smoking Index (HSI) by race/ethnicity.

| **Combination** | **Group** | **n** | **Agreement (%)** | **Expected agreement (%)** | **Kappa** | **SE** | **Bootstrap CI**^a^ |
| --- | --- | --- | --- | --- | --- | --- | --- |
| 1 | White, Non-Hispanic | 4635 | 88.24 | 70.00 | 0.60 | 0.012 | 0.53-0.57 |
|  | Black, Non-Hispanic | 709 | 89.26 | 72.43 | 0.62 | 0.032 | 0.55-0.66 |
|  | Hispanic, Any Race | 372 | 90.86 | 71.31 | 0.68 | 0.047 | 0.60-0.73 |
|  | Asian, Non Hispanic | 56 | 91.07 | 74.68 | 0.64 | 0.125 | 0.43-0.82 |
|  | Native American, Non Hispanic | 18 | 86.11 | 70.99 | 0.52 | 0.164 | 0.16-0.87 |
|  | N/A or Other | 144 | 89.24 | 69.57 | 0.65 | 0.063 | 0.49-0.73 |
| 2 | White, Non-Hispanic | 4635 | 75.32 | 63.51 | 0.33 | 0.011 | 0.30-0.35 |
|  | Black, Non-Hispanic | 709 | 86.18 | 80.57 | 0.29 | 0.026 | 0.19-0.38 |
|  | Hispanic, Any Race | 372 | 88.44 | 82.98 | 0.32 | 0.038 | 0.17-0.47 |
|  | Asian, Non Hispanic | 56 | b | - | - | - | - |
|  | Native American, Non Hispanic | 18 | 77.78 | 62.96 | 0.40 | 0.185 | 0.01-0.85 |
|  | N/A or Other | 144 | 78.47 | 68.54 | 0.32 | 0.061 | 0.16-0.48 |
| 3 | White, Non-Hispanic | 4635 | 92.00 | 80.70 | 0.59 | 0.014 | 0.55-0.62 |
|  | Black, Non-Hispanic | 709 | 97.60 | 93.06 | 0.65 | 0.037 | 0.49-0.79 |
|  | Hispanic, Any Race | 372 | 96.24 | 93.25 | 0.44 | 0.052 | 0.16-0.67 |
|  | Asian, Non Hispanic | 56 | b | - | - | - | - |
|  | Native American, Non Hispanic | 18 | 94.44 | 84.57 | 0.64 | 0.220 | 0.00-1.00 |
|  | N/A or Other | 144 | 95.14 | 84.09 | 0.70 | 0.081 | 0.44-0.89 |
| 4 | White, Non-Hispanic | 4635 | 87.68 | 55.49 | 0.72 | 0.015 | 0.70-0.75 |
|  | Black, Non-Hispanic | 709 | 86.60 | 69.56 | 0.56 | 0.037 | 0.48-0.63 |
|  | Hispanic, Any Race | 372 | 92.74 | 74.86 | 0.72 | 0.051 | 0.62-0.82 |
|  | Asian, Non Hispanic | 56 | 91.07 | 85.08 | 0.65 | 0.125 | -0.02-1.00 |
|  | Native American, Non Hispanic | 18 | 88.89 | 55.56 | 0.75 | 0.229 | 0.31-1.00 |
|  | N/A or Other | 144 | 86.11 | 58.07 | 0.67 | 0.082 | 0.55-0.80 |
| 5 | White, Non-Hispanic | 4635 | 78.21 | 62.48 | 0.42 | 0.012 | 0.39-0.45 |
|  | Black, Non-Hispanic | 709 | 83.36 | 77.56 | 0.26 | 0.026 | 0.18-0.35 |
|  | Hispanic, Any Race | 372 | 87.63 | 81.32 | 0.34 | 0.039 | 0.20-0.47 |
|  | Asian, Non Hispanic | 56 | 91.07 | 87.88 | 0.26 | 0.090 | 0.00-0.78 |
|  | Native American, Non Hispanic | 18 | 72.22 | 64.81 | 0.21 | 0.142 | 0.00-0.69 |
|  | N/A or Other | 144 | 79.17 | 64.84 | 0.41 | 0.067 | 0.25-0.57 |

*Note.* CI= Confidence Intervals; ^a^Bias corrected with 1000 samples.

1. HSI Low 0–1, Moderate 2–4, High 5–6 vs. FTCD Low <= 3, Moderate 4–7, High> 7.

2. HSI Low–Moderate <4, High>=4 versus FTCD Low–Moderate<8, High>=8.

3. HSI Low–Moderate<=4, High>4 versus FTCD Low–Moderate<8, High>=8.

4. HSI Low–Moderate<4, High>=4 versus FTCD Low–Moderate<6, High>=6.

5. HSI Low–Moderate<=4, High>4 versus FTCD Low–Moderate<6, High>=6.

^b^ No cases for high dependence, thus concordance is not calculated.

Table s10. Correlation and regression analyse to analyse the concordance among the continuous measures of the Fagerström Test for Cigarette Dependence (FTCD) and Heaviness of Smoking Index (HSI) global scores separately by sex at birth.

|  | Global | | Males | | Females | |
| --- | --- | --- | --- | --- | --- | --- |
| Statistical method | Coeff. | CI | Coeff. | CI | Coeff. | CI |
| Pearson | .89 | .89-.90 | .89 | .88-.89 | .89 | .88-.90 |
| Spearman | .88 | .87-.89 | .88 | .87-.89 | .89 | .88-89 |
| Lin correlation-concordance coefficient (rho_c) | .61 | .60-.62 | .61 | .59-.62 | .61 | .59-.62 |
| Regression |  |  |  |  |  |  |
| B_FTCD_ | .60 | .60-.61 | .62 | .61-.63 | .59 | .58-.60 |
| β_FTCD_ | .89 |  | .89 |  | .89 |  |
| Deming regression |  |  |  |  |  |  |
| FTCD | .65 | .64-.66 | .67 | .66-.68 | .63 | .62-.64 |
| Intercept | -.09 | -.12- -.06 | -.16 | -.21--.10 | -.03 | -.07-.01 |

Table s11. Correlation and regression analyse to analyse the concordance among the continuous measures of Fagerström Test for Cigarette Dependence (FTCD) and Heaviness of Smoking Index (HSI) by race/ethnicity.

|  | White, Non-Hispanic | | Black, Non-Hispanic | | Hispanic (Any Race) | | Asian, Non-Hispanic | | Native American, Non-Hispanic | | N/A or Other | |
| --- | --- | --- | --- | --- | --- | --- | --- | --- | --- | --- | --- | --- |
| Statistical method | Coeff. | CI | Coeff. | CI | Coeff. | CI | Coeff. | CI | Coeff. | CI | Coeff. | CI |
| Pearson | .89 | .88-.90 | .87 | .85-.89 | .89 | .87-.91 | .85 | .76-.91 | .91 | .78-.97 | .87 | .82-.90 |
| Spearman | .88 | .87-.89 | .86 | .84-.88 | .89 | .87-.91 | .85 | .75-.91 | .89 | .72-.96 | .87 | .82-.91 |
| Lin correlation-concordance coefficient (rho_c) | .60 | .59-.61 | .58 | .56-.60 | .64 | .60-.68 | 0.51 | 0.40-0.62 | .58 | .39-.67 | .58 | .51-.65 |
| Regression |  |  |  |  |  |  |  |  |  |  |  |  |
| B_FTCD_ | .60 | .59-.61 | .58 | .56-.60 | .61 | .58-.64 | .60 | .47-.65 | .54 | .41-.67 | .62 | .56-.68 |
| β_FTCD_ | .89 |  | .87 |  | .89 |  | .85 |  | .91 |  | .87 |  |
| Deming regression |  |  |  |  |  |  |  |  |  |  |  |  |
| FTCD | .64 | .63-.65 | .63 | .61-.65 | .65 | .62-.68 | .61 | .51-.71 | .56 | .46-.66 | .69 | .62-.75 |
| Intercept | -.02 | -.07-.01 | -.14 | -.23--.05 | -.24 | -.35--.13 | -.18 | -.50-.15 | .22 | -.33-.76 | -.35 | -.62--.07 |

**Figure s1.** Bland-Altman plot assessing agreement between Fagerström Test for Cigarette Dependence (FTCD) and Heaviness of Smoking Index (HSI) scores by sex at birth. The black dashed line represents the mean difference between FTCD and HSI. The grey shaded area depicts the 95% limits of agreement. Data point size correlates with the number of cases, indicating variability in the differences between the two measures. A solid line at axis-y = 0 indicates no difference between both measures.

|  |
| --- |
|  |
|  |

**Figure s2.** Bland-Altman plot assessing agreement between Fagerström Test for Cigarette Dependence (FTCD) and Heaviness of Smoking Index (HSI) scores by race/ethnicity. The black dashed line represents the mean difference between FTCD and HSI. The grey shaded area depicts the 95% limits of agreement. Data point size correlates with the number of cases, indicating variability in the differences between the two measures. A solid line at axis-y = 0 indicates no difference between both measures.

1. Cinciripini PM, Karam-Hage M, Kypriotakis G, Robinson JD, Rabius V, Beneventi D, et al. Association of a Comprehensive Smoking Cessation Program With Smoking Abstinence Among Patients With Cancer. JAMA Network Open. 2019 Sep 27;2(9):e1912251. [↑](#footnote-ref-2)
